# Dual square split ring enclosed spiral shaped hybrid metamaterial resonator with size miniaturisation for microwave wireless applications

**DOI:** 10.1038/s41598-022-11993-0

**Published:** 2022-05-16

**Authors:** Air Mohammad Siddiky, Mohammad Rashed Iqbal Faruque, Sabirin Abdullah, Mohammad Tariqul Islam, Mayeen Uddin Khandaker, K. S. Al-Mugren

**Affiliations:** 1 Space Science Center (ANGKASA), Universiti Kebangsaan Malaysia, 43600 Bangi, Selangor Malaysia; 2 Department of Electrical, Electronic and Systems Engineering, Universiti Kebangsaan Malaysia, 43600 Bangi, Selangor Malaysia; 3 Centre for Applied Physics and Radiation Technologies, School of Engineering and Technology, Sunway University, 47500 Bandar Sunway, Selangor Malaysia; 4 Department of Physics, College of Science, Princess Nourah bint AbdulRahman University, 84428, Riyadh, 11671 Saudi Arabia

**Keywords:** Electrical and electronic engineering, Techniques and instrumentation

## Abstract

In this research work, the development of the metamaterial unit cell is used to investigate multifunctional characteristics, exhibit preferable and capable adjustability, reconfigurable by changing the phase response of applied electromagnetic wave. This proposed metamaterial unit cell is analysed by modifying the geometric design of the metallic structure which mitigates the design to reduce the cost for the commercialisation. The resonant frequencies are located from 1.87, 2.55, 4.32, 5.46 GHz. The interaction with the electric field and magnetic field exhibit the polarisation in both planes which enhances the left handed characteristics. The field distribution of electric, magnetic, and surface current is presented with vector fields in different planes to observe the polarisation state. Different thicknesses of dielectric material are utilised to observe the impact of time varying electric and magnetic fields through the proposed metamaterial. The different substrate materials are described the degree of freedom for the implementation in different fields within the functional microwave frequency range.

## Introduction

Metamaterial exhibits a strong localization and enhances the fields which improve the compatibility for different satellite band applications significantly. The exotic properties will assess in different forms of design with improved characteristics of the bandwidth, frequency selectivity and physical size. The periodical arrangement of the artificial subwavelength inclusion, the dimension of the unit cell, thickness and metal strips are essential parameters to obtain the selective operational bandwidth. For the implementation of the external electric and magnetic field, the resonant frequency is obtained due to the inductive and capacitive effect of the metal-dielectric based resonator. The electromagnetic properties can be controlled by tailoring the geometric dimension and design of the metallic structure. The unusual electromagnetic properties are introduced by the addition of split ring resonator in the subwavelength region which exhibits simultaneous negative permittivity, negative permeability and negative refractive index at a certain frequency range. Different categories can be defined such as ENG metamaterial for single negative permittivity, MNG metamaterial for single negative permittivity and DNG metamaterial for both negative permittivity and permeability^1–7^. Recent advents in the wireless communication systems have enormously raised the demand in various millimetre-submillimetre based microwave applications such as energy harvesting, absorber, SAR reduction, power splitter, microwave imaging, chemical sensing, lens, coding, enhancement of the antenna bandwidth and directivity, etc.^7–16^.

In wireless communication technologies, the metamaterial based research is increasing where the connectivity to create a network and sensing data remotely has become an important factor in recent years. An electrically tunable metamaterial based decagonal shaped split ring resonator integrated with varactor diode. The scattering characteristics were studied with different array cells and exhibit tunable left handed characteristics from 2.71 to 5.5 GHz which can be applicable for different microwave wearable applications^17^. An open interconnected split ring resonator was loaded for the compact double notch microstrip bandstop filter. Additional notch band act as a RLC circuit and can be controlled by adding more notch for the different frequency responses^18^. For the precise measurement of the real and imaginary part of the permittivity of the solid dielectric, an advanced investigation was carried out using a modified split ring resonator. A mathematical model was developed on a curve fitting tool to obtain the relation for the real and imaginary part of the permittivity with the sample’s thickness and quality factor^19^. Reconfigurable terahertz metamaterial using split ring resonator exhibit multifunctional electromagnetic properties such as polarisation switching, resonance shifting, EIT switching, multiband to single band switching and the suggested metamaterial analysed with different orientation in transverse electric and magnetic modes^20^. Near-zero refractive index based metamaterial with symmetrically arranged split ring resonator which was integrated into the proposed monopole antenna can operate over a wider bandwidth from the 3.08 to 14.1 GHz for ultra-wideband applications^21^. The wireless passive temperature sensor was designed with double split ring resonator on an alumina ceramic substrate which was fabricated using micromechanical and screen printing technology and measured resonant frequency obtained at 2.42 GHz for a wider range of applications in military, aerospace, RFID tag, etc.^22^. Minkowski-like fractal geometry was applied to design a microstrip patch antenna with a composite right/left handed transmission line for dual band satellite applications. This miniaturised antenna provides high gain of 3.8 dB and 6.2 dB with an omnidirectional radiation pattern at 4.4 and 6.1 GHz^23^. A shared-aperture phased-array antenna produced wide beam with a triangular placement to avoid grating lobes, where a mushroom shaped metamaterial structure was proposed to reduce the array spacing^24^. A wideband circularly polarise antenna with composite metamaterial was established which was based on a modified co-planar waveguide feeding technique for small satellite applications. The circular polarisation obtained by placing two transmission lines, where the metasurface structure enhanced bandwidth, gain and the axial ratio^25^. Flexible wearable ultra-wideband antenna with metamaterial structure was designed for wireless body network and tumor detection with a diameter of 4 mm by breast imaging. Six modified metamaterial unit cells improved the gain, bandwidth, directivity and reduce the specific absorption rate for the proposed antenna^26^. A square shaped EBG based printed antenna with Hilbert-shaped metamaterial array using nickel oxide polymerized palm fibre for RF energy harvesting and exhibit improved bandwidth with high gain about 4.56 dB and 7.38 dB at 5.8 and 8 GHz, respectively^27^.

In this study, we have presented a square split ring resonator with double split rings and a spiral resonator for S and C band applications with four resonant frequencies. The combination of these three split ring resonators enhances the polarisation for the external electric and magnetic fields in the parallel and transverse planes. The metallic layer of the proposed metamaterial unit cell is printed on the FR-4 substrate with the dimension 9 × 9 × 1.6 mm^3^. The proposed design produce resonant frequencies with the strong accumulation of the electromagnetic field and exhibit reconfigurability with different phase response. Moreover, the proposed design shows high compactness where the effective medium ratio is 17.

## Suggested metamaterial unit cell and equivalent circuit model

**Figure 1:**
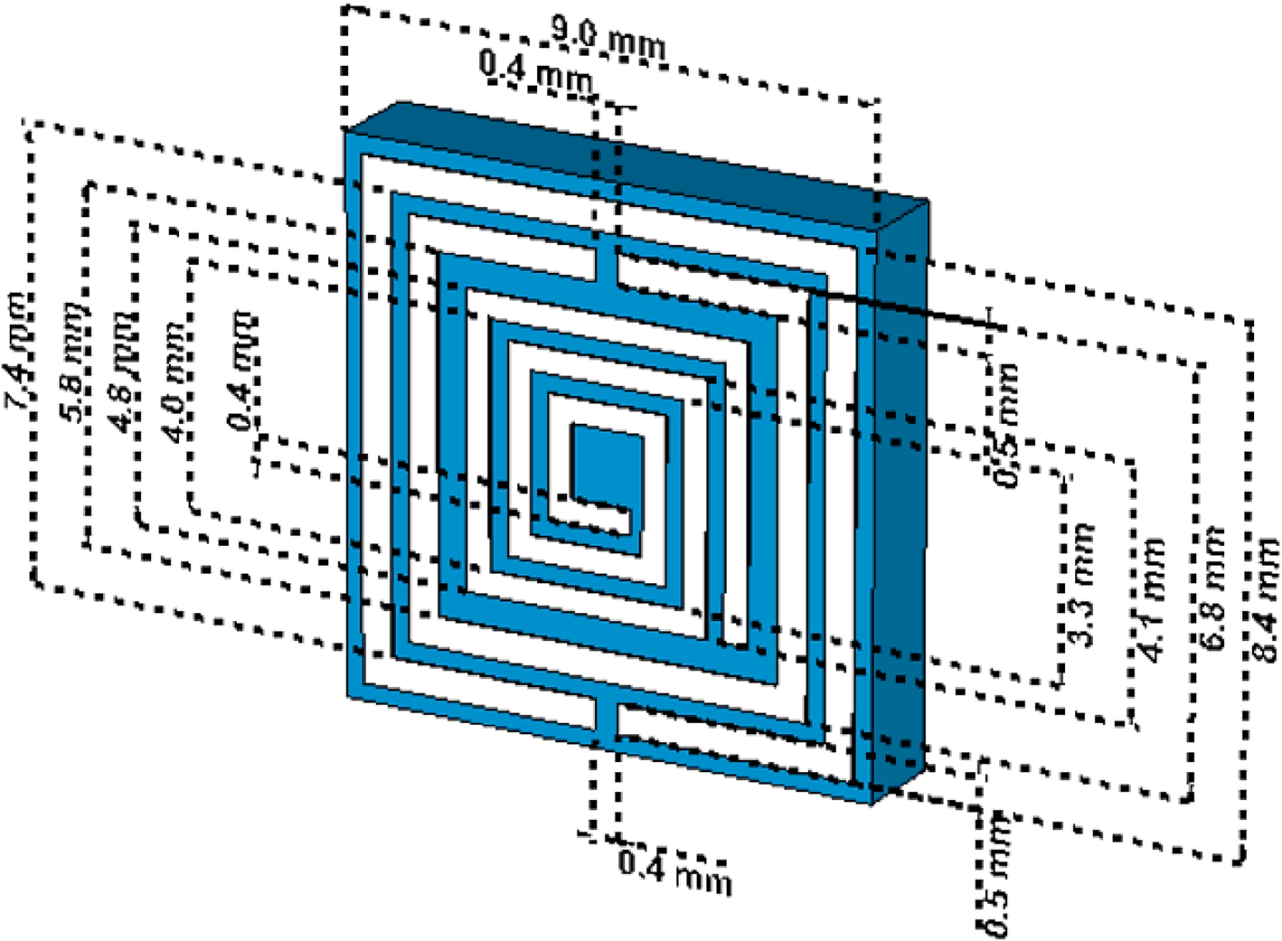
Proposed metamaterial unit cell with the dimensional specification.

**Figure 2:**
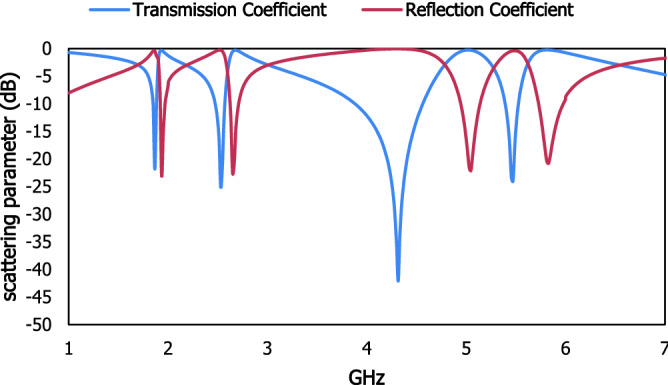
Scattering result of the proposed metamaterial unit cell.

Three discrete metallic strips are introduced for the proposed metamaterial unit cell and schematically geometric specifications are shown in Fig. 1, scattering result in Fig. 2 and dimensional specification in Table 1. The split ring type geometric design of the metallic layer on the dielectric medium has exhibited the enhancement of the magnetic susceptibility for applied transverse electromagnetic wave. The combination of two square split ring resonator and one spiral resonator is improved the electric and magnetic polarisation which enhance the negative electric and magnetic susceptibility. The outer split ring resonator is loaded on the dielectric FR-4 substrate extended from 3.7 to 4.2 mm with the split 0.3 mm and the inner ring is deployed with the distance of 0.9 mm where the width of the metallic arm is 0.5 mm. The two split ring resonator is introduced the inductive and capacitive response with the interaction of the time varying electric and magnetic field. The split portion of the inner ring has a width of 0.3 mm. spiral loop metallic resonator is introduced on the middle part of the design with a distance of 0.45 mm from the inner split ring resonator. The complementary ring resonator is used as the outer metallic loop with an inner spiral type resonator which is introduced to the resonant frequencies. The combination of the spiral shaped metallic layer and two split ring resonator enhances the magnetic coupling with the subwavelength based metamaterial structure. Inductive properties of metallic layer dominate over the capacitive value of dielectric substrate in the higher frequency region. The metallic layer of copper exhibit the inductive response, whereas the dielectric substrate and the space between the metallic arm, are responsible for the capacitive response^28^. This subwavelength based metamaterial based resonator act as an LC resonant circuit and the resonant frequency can be written as,

**Table 1:** Dimensional specification for the proposed metamaterial unit cell.

	The split distance in the outer ring	The split distance in the inner ring	Space between two split rings	Width of the split ring
mm	0.4	0.4	0.3	0.5
	Width of the spiral shape metallic arm	Space between the inner spiral shape patch	Conducting material thickness	Dielectric material thickness
mm	0.4	0.4	0.035	1.6

1 $$f=\frac{1}{2\pi \sqrt{LC}}$$

**Figure 3:**
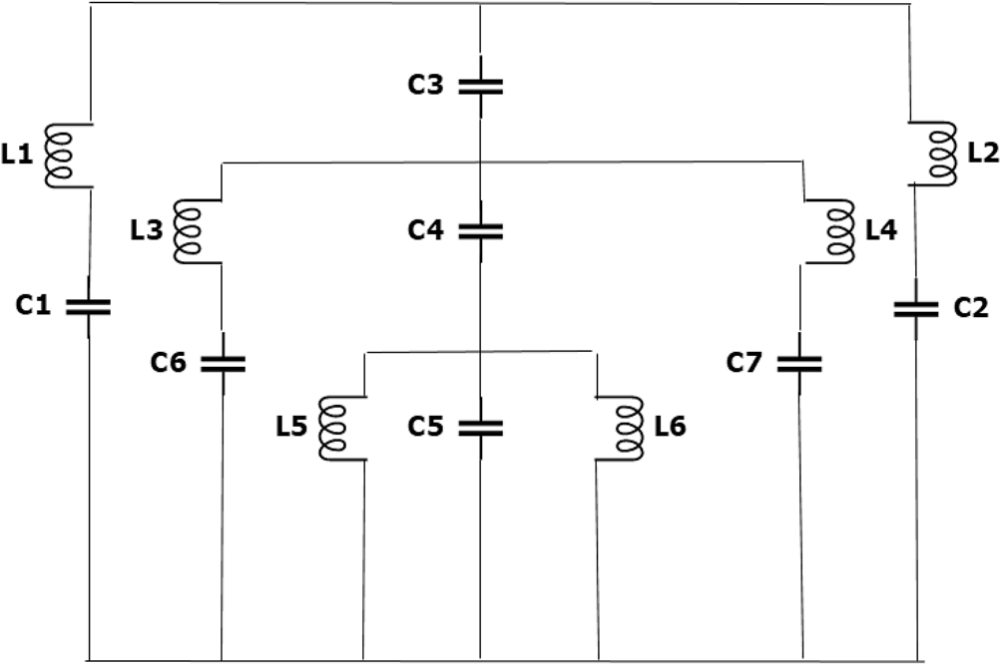
Equivalent circuit model.

**Figure 4:**
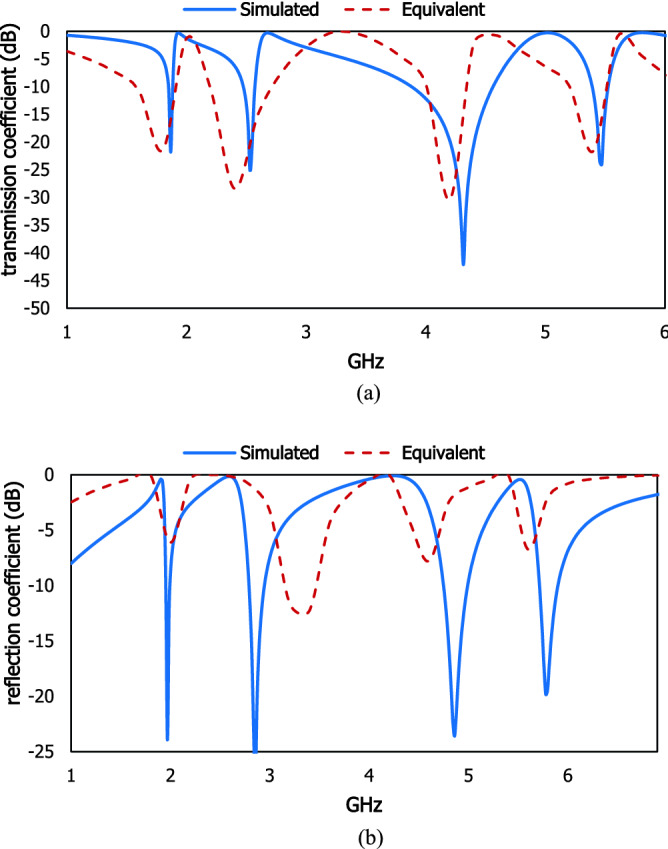
(**a**) Transmission coefficient and (**b**) reflection coefficient for simulated and equivalent circuit model.

For the proposed metamaterial unit cell the lumped elements are presented as (L1, C1), (L2, C2) for the first loop of split ring, (L3, C5), (L4, C7) for the inner square split ring, parallel branch (L5, C6, L6) for open loop spiral shape resonator and C3, C4 are used for the capacitance between the metallic arm and dielectric substrate. The equivalent circuit is drawn in Fig. 3 and the corresponding result in Fig. 4 is validated by ADS software. When the two parallel plates are placed on the dielectric substrate carry the same current in the same direction at a distance S from the centre, the self and mutual inductance are introduced in parallel where each wire adds up in series. For single loop metallic strip, the total inductance can be expressed as,

2 $${L}_{(nH)}=1.257\times {10}^{-3} \mathrm{a}\left[\mathrm{ln}\left(\frac{a}{w+t}\right)+0.078\right]{K}_{g}$$

where, a is the radius of the metallic loop.

For spiral loop resonator,

3 $${L}_{(nH)}=0.03937{\frac{{a}^{2}{n}^{2}}{8a+11c}K}_{g}$$

where a = $\frac{{D}_{0}+{D}_{i}}{4}$; c = $\frac{{D}_{0}-{D}_{i}}{2}$; D_0_ is the outer radius and D_i_ is the inner radius of the spiral loop.

$${K}_{g}=0.57-0.145 \mathrm{ln}\frac{W}{h}$$

W is the width of the metallic strip and h is the thickness of the dielectric substrate.

The total capacitance for a single loop can be expressed as:

4 $$\mathrm{C}\left(\mathrm{pF}\right)=\frac{{\varepsilon }_{e}{10}^{-3} K\left(k\right)}{18\pi {K}^{^{\prime}}\left(k\right)}(N-1)l$$

where, K = ${tan}^{2}( \frac{a\pi }{4b}$); a = $\frac{w}{2}; b= \frac{w+s}{2} ;$ W is the width of the conductor and S is the space between two parallel plates. ${\varepsilon }_{e}$ is the effective dielectric constant. The capacitance effect between the metal to the dielectric substrate, two parallel plates and fringing field is also taken into account where the values may be varied from 0.1 to 10 pF and the inductance value can be varied from 0.1 to 20 nH for different factor such as length, turn ratio, width of metallic strip.

## Selection of the proposed design

**Figure 5:**
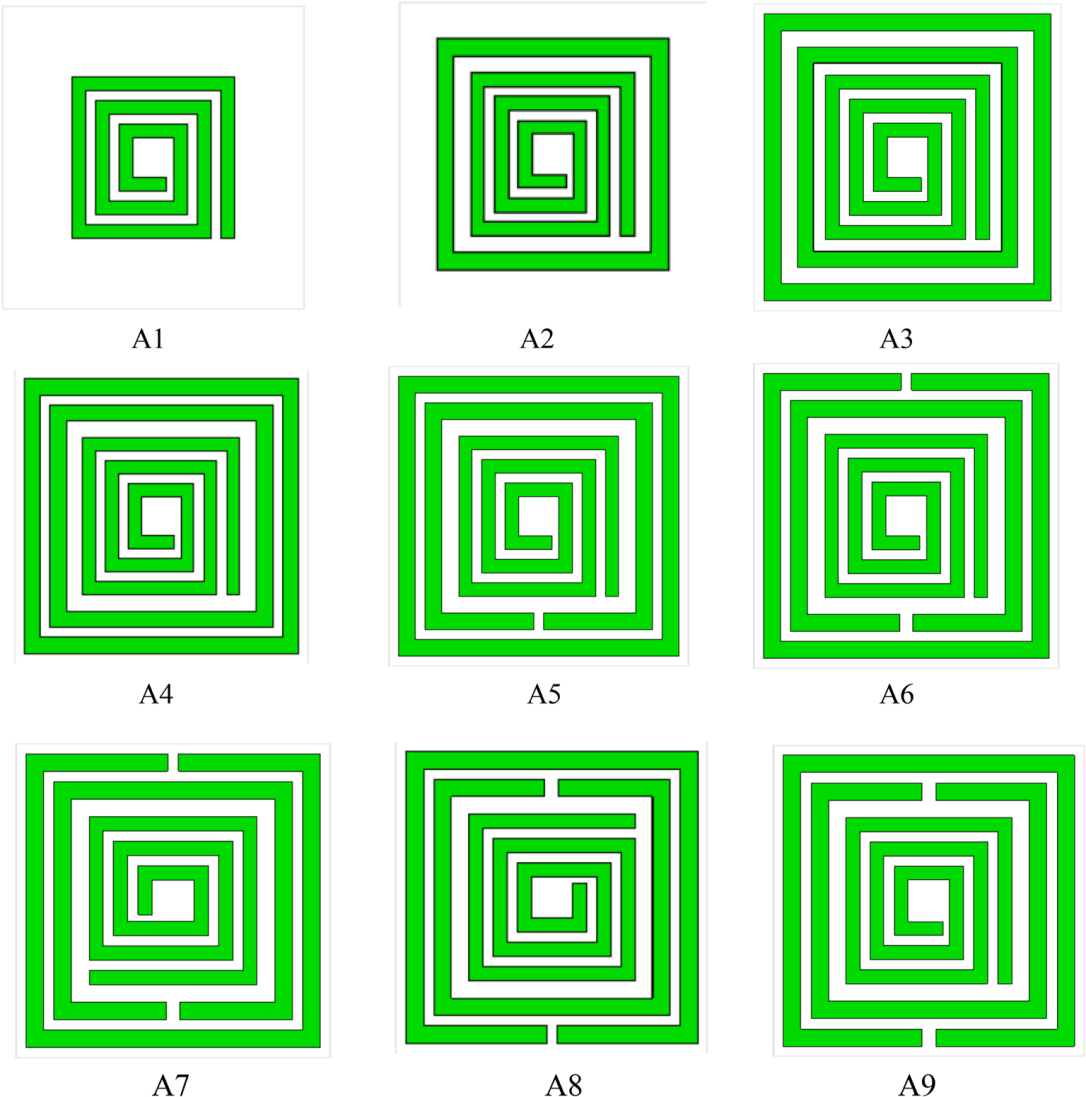
Design selection for the compact metamaterial resonator.

**Figure 6:**
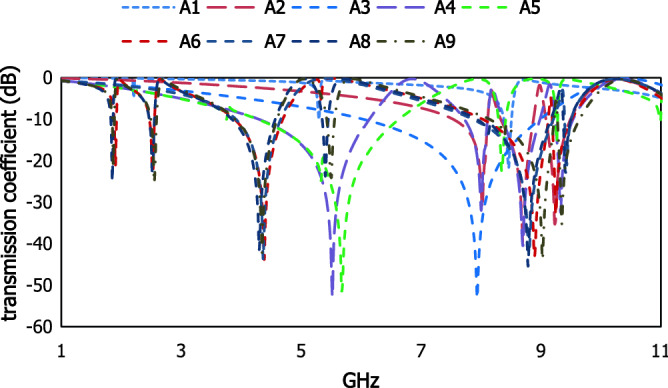
Results for different designs.

The optimization for the proposed square split ring resonator is taken into consideration by selecting proper physical parameters and the coupling effect between the resonators. Dimensional variation in the conducting layer makes this artificial structure resonate at different frequencies for the required band applications. The metamaterial design selection stage is presented in Fig. 5, corresponding result in Fig. 6 and data specification in Table 2. A spiral open loop metallic arm is deployed on the dielectric substrate producing resonant frequency at 8.46 GHz, for design 1 with the bandwidth from 8.24 to 8.57 GHz. In design 2, a closed loop resonator is added with design 1 which exhibits three resonant frequencies at 7.96, 8.66, 9.16 GHz. Two closed loop metallic arms are enclosed an open loop metallic arm in design 3 and the resonant frequencies are shifted to lower frequency region at 5.4, 8.13, 8.95 GHz. Introducing a more metallic layer is increased the inductance to change the scattering properties. The dimension of the inner split ring in design 4 is extended to reduce the space between the two split ring resonators and four resonant frequencies are obtained at 5.47, 7.93, 8.63, 9.23 GHz due to reduction in space and rise in inductive value. By removing a metallic part with the dimension 0.4 mm from the inner metallic loop is presented in design 5 which is introduced the LC branch circuit and exhibit three resonant frequencies. In addition, the outer metallic closed loop arm is cut away in design 6 and the combination of the two split ring resonator is increased the parallel effect of the lumped elements with the inner spiral shape open loop conducting layer. In design 7, the inner spiral-shaped metallic layer rotates in a clockwise direction with 90 degrees where the end of the open loop metallic arm gets aligned with the inner split ring. In design 8, the outer two split ring resonator and inner spiral shape metallic arm are rotated with 180 degrees and the resonant frequencies are shifted to the lower frequency region. In the proposed design A9 exhibits four resonant frequencies at 1.87, 2.55, 4.32, 5.46 GHz within 1–6 GHz. This arrangement of the metallic strip with the applied electromagnetic wave changes the impedance effect due to the portion of the interacted electric and magnetic field component where the other design exhibit resonant frequencies to the higher frequency region and reduce the compatibility for the compact artificial structure.

**Table 2:** Data specification for the different unit cell designs.

Design	Bandwidth (GHz)	Resonant frequency within 1–10 GHz
A1	8.24–8.57	8.46
A2	7.48–8.04, 8.24–8.8, 9.0–9.5	7.96, 8.66, 9.16
A3	4.2–6.1, 8.04–8.13, 8.45–9.17	5.4, 8.13, 8.95
A4	4.28–6.1, 7.6–8.04, 8.27–8.9, 9.13–9.48	5.47, 7.93, 8.63, 9.23
A5	4.19–6.64, 8.18–8.35, 9.18–9.24	5.63, 8.27, 9.2
A6	1.87–1.95, 2.43–2.57, 3.85–4.69	1.9, 2.52, 4.37
A7	3.88–4.68, 7.8–9.2, 9.32–9.45	4.37, 8.74, 9.36
A8	1.87–1.89, 2.46–2.56, 3.83–4.70, 7.83–9.17, 9.35–9.45	1.88, 2.5, 4.38, 8.77, 9.38
A9	1.84–1.86, 2.45–2.58, 3.85–4.57, 5.37–5.56, 7.98–9.1, 9.17–9.54	1.87, 2.55, 4.32, 5.46, 8.9, 9.25

## Methodology

A boundary condition is applied for the perfect interaction of electromagnetic (EM) wave energy with the subwavelength resonator. A transverse electromagnetic wave within the microwave frequency range is energized from the waveguide port. The dimension of the waveguide port, the distance between the metamaterial based resonator and waveguide port are defined for the proper interaction with the applied electromagnetic wave. A tetrahedral mesh using the FEM technique is utilized for the numerical simulation of the proposed design to observe the scattering properties for applied specific frequency region in CST microwave studio 2017^29^. For a defined boundary condition a transverse electromagnetic wave is travelled through the dielectric substrate at Z direction, where the proposition for magnetic field functioning along Y-axis and electric field along X-axis. Time varying magnetic field generated electric field, where the changing electric field generates a magnetic field. The interference pattern of the electromagnetic wave determines the wave characteristics of the wave propagation through the medium. The characterization of the metal-dielectric based resonator is related to the interaction of the time varying electric and magnetic field, exhibit the unusual scattering properties for the continuous finite slab. The effective parameters are addressed the polarisation effect of the metal-dielectric based artificial subwavelength inclusion, the degree of magnetization of the conducting layer for different geometric orientations and the propagation of the electromagnetic wave through the resonator. Nicolson–Ross–Weir^30^ method is implemented in MATLAB software to characterise the effective parameters such as effective permittivity, effective permeability and refractive index from the transmission coefficient (S_21_) and reflection coefficient (S_11_). The scattering parameters of the proposed SRR design can be written as follows:

6 $$\mathrm{Relection} \; \mathrm{coefficient}, {S}_{11}=\frac{(1-{\Gamma }^{2})\mathcal{Z}}{1-{\Gamma }^{2}{\mathcal{Z}}^{2}}$$

7 $$\mathrm{Transmission \; coefficient}, {S}_{21}=\frac{(1-{\mathcal{Z}}^{2})\Gamma }{1-{\Gamma }^{2}{\mathcal{Z}}^{2}}$$

The equation of the effective permittivity (${\varepsilon }_{r}$) is defined as:

8 $${\varepsilon }_{r}=\frac{2}{j\pi fd}\times \frac{(1-{S}_{21}-{S}_{11})}{(1+{S}_{21}+{S}_{11})}$$

The equation of the effective permeability (μ_r_) can be expressed as:

9 $${\mu }_{r}= \frac{2}{j\pi fd}\times \frac{(1-{S}_{21}+{S}_{11})}{(1+{S}_{21}-{S}_{11})}$$

where Z = $$\sqrt{\frac{{(1+{S}_{11})}^{2}-{{S}_{21}}^{2}}{{(1-{S}_{11})}^{2}-{{S}_{21}}^{2}}}$$

The engineered unit cell geometry follows the periodic arrangement and the metallic loop with a certain dimension exhibits negative permittivity and permeability. The artificial magnetism properties with negative permittivity at a higher frequency region introduce the refractive index which describes the propagation of the applied electromagnetic wave through the medium.

Refractive index can be expressed as,

10 $$\eta =\frac{1}{{k}_{o}d}\left\{\left[\left[\mathrm{ln}({e}^{jn{k}_{o}d})\right]\right]+2m\pi -j\left[\mathrm{ln}({e}^{jn{k}_{o}d})\right]\right\}$$

where, $${e}^{jn{k}_{o}d}= \frac{{S}_{21}}{1-{S}_{11}\frac{Z-1}{Z+1}}$$

## Results and discussion

**Figure 7:**
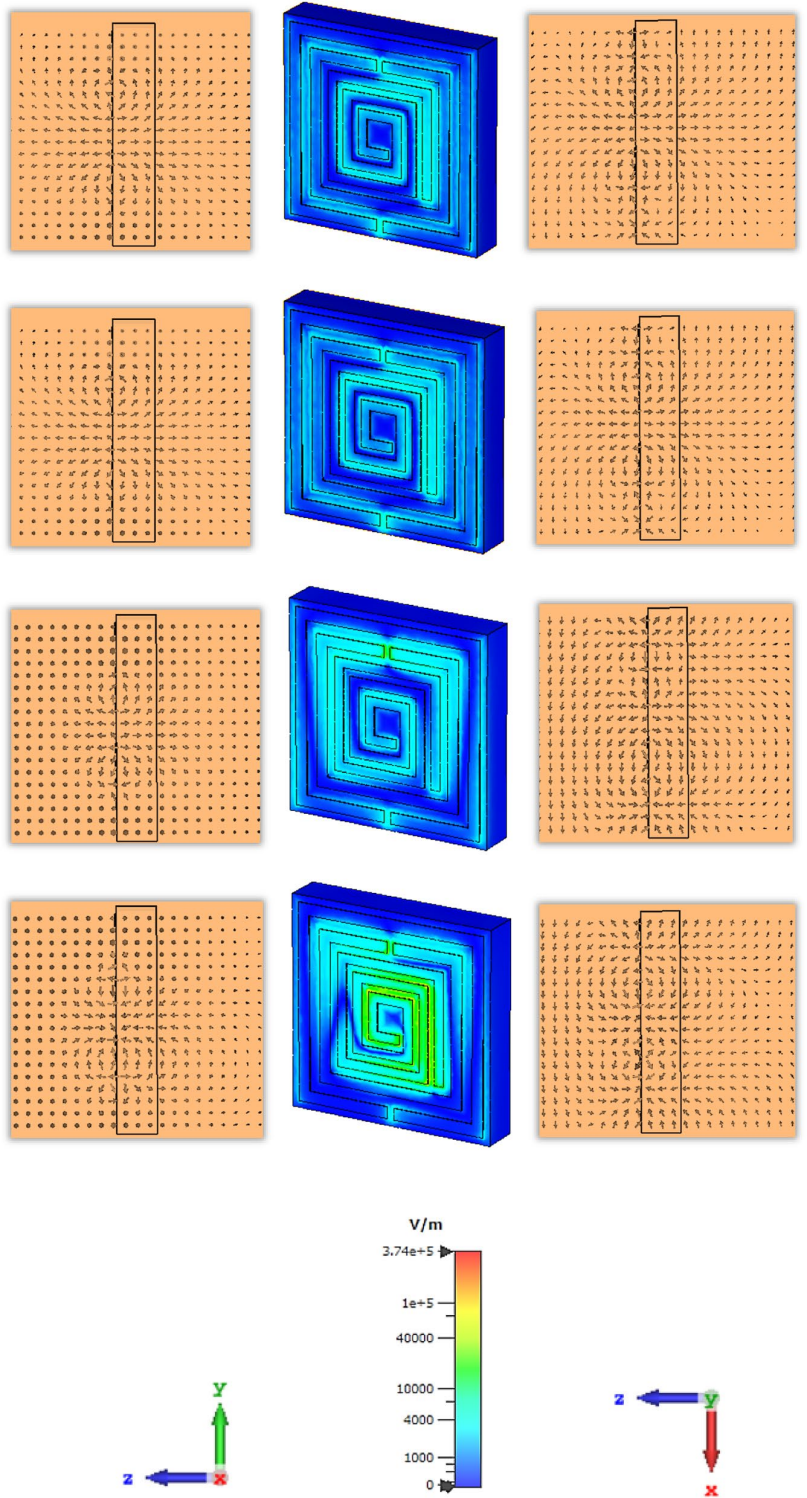
Electric field distribution in row-wise ascending from the top order for different resonant frequencies of 1.87, 2.55, 4.32, 5.46 GHz successively.

**Figure 8:**
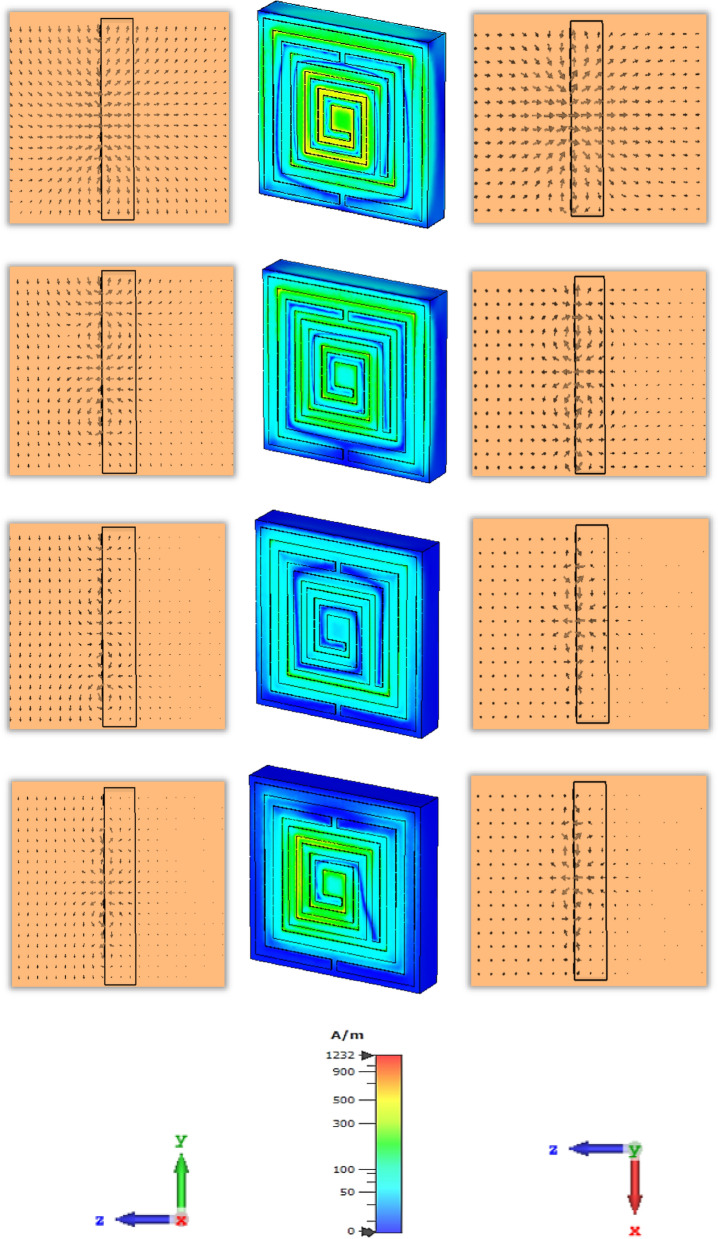
Magnetic field distribution in row-wise ascending from the top order for different resonant frequencies of 1.87, 2.55, 4.32, 5.46 GHz successively.

The scattering parameters for the subwavelength based metamaterial resonator is significantly dependent on the applied direction of the electric field and magnetic field. The molecules in the dielectric substrate have experienced a force to be aligned with the applied electrical field and produce an electric dipole moment to change the potential energy. The applied magnetic field is the enhanced angular momentum of the charged molecules which depends on the frequency and field strength. For this proposed metamaterial resonator the electric field is applied in the X-direction and the magnetic field in the transverse plane. The vector field distribution for the electric field and magnetic field are presented in Figs. 7 and 8. The first resonant frequency at 1.9 is exhibited polarisation in the X plane, follow the anticlockwise direction in the left outer metallic split arm and clockwise direction in the right inner metallic split arm. Meanwhile, the electric field is polarised in the Y plane due to the split ring resonator which is introduced exotic properties of the subwavelength based artificial inclusion of the metamaterial. The electric field is polarized in the X plane for the second resonant frequency follows the opposite direction of the first resonant frequency, where the polarisation takes place in the Y direction around the outer and inner metallic split ring. For the third resonant frequency, the polarisation for the external electric field has occurred around the inner metallic layer for the X plane, where the polarisation in the Y plane is exhibited around two split rings and an inner spiral metallic design. Moreover, electric field polarisation is followed the outward direction from the end of the spiral resonator and inward direction from the middle portion of the design in the X plane, where the vector field of electric polarisation is followed the outward direction from the left outer ring and inward direction from the inner spiral resonator.

The magnetic resonance is produced due to the polarisation of the external magnetic field along the parallel plane and transverse plane due to the geometric configuration of split ring resonator. At the first resonant frequency, the magnetic field is polarized around the middle split ring resonator, where the vector field is followed the anticlockwise direction in the upper part and clockwise direction from the middle split arm to the lower part of the split ring. The magnetic field is distributed around the outer split ring resonator and inner spiral shape metallic, which enhances the polarisation for the applied excitation along the horizontal and vertical plane of the proposed metamaterial unit cell at the second resonant frequency. For the third resonant frequency, the vector presentation for magnetic polarisation exhibit the intensity higher around the surface of the conducting layer, where the polarisation for time varying magnetic field shows low intensity beside the dielectric substrate end due to the interaction with the lower EM wavelength and the lack of conducting layer. At the fourth resonant frequency, the magnetic field is distributed over free space. The polarized field vector is distributed in both planes for the applied time varying magnetic field which increases the diamagnetism effect within the subwavelength region.

**Figure 9:**
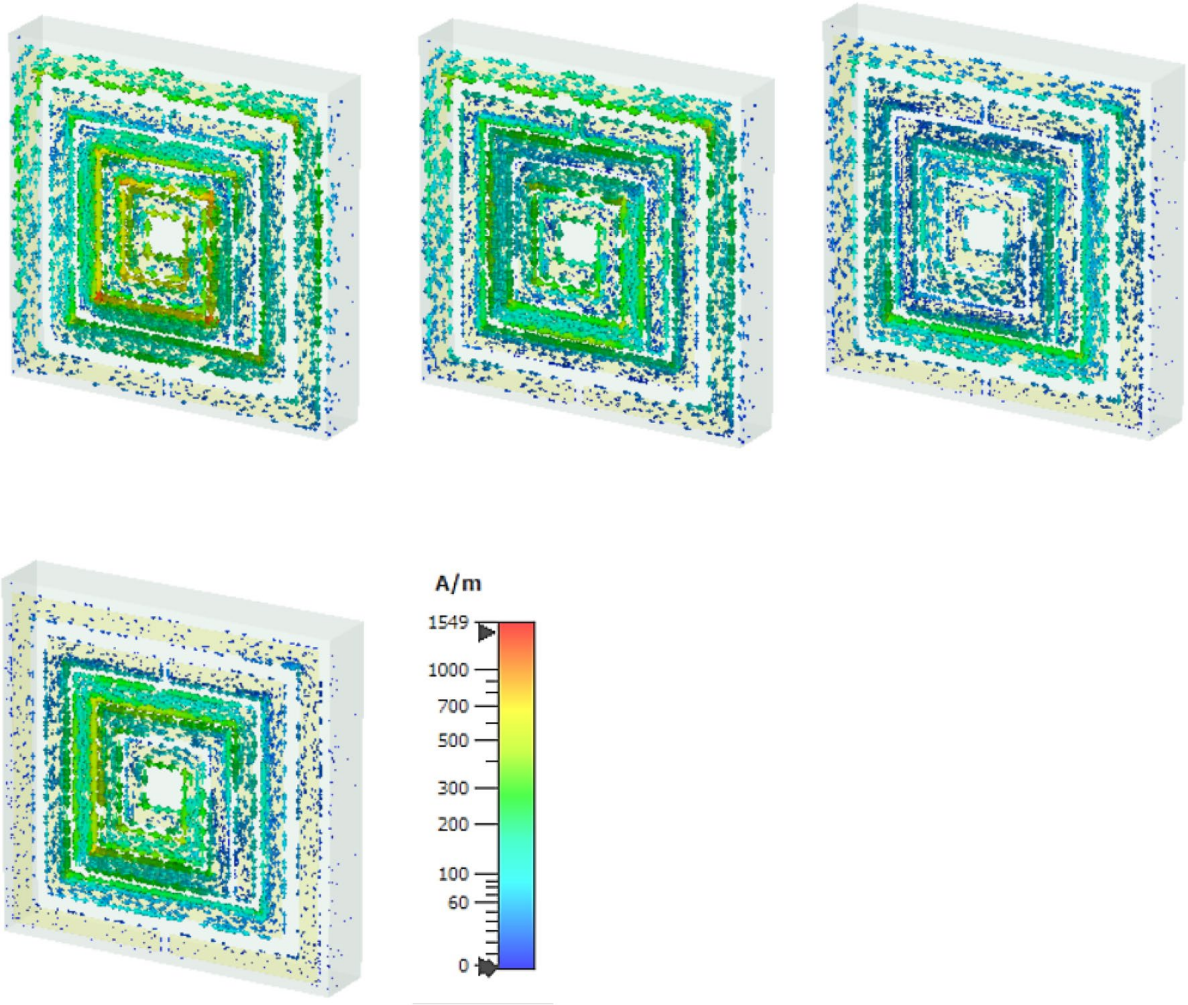
Surface current distribution in row-wise ascending from the top order for different resonant frequencies of 1.87, 2.55, 4.32, 5.46 GHz successively.

The surface current distribution for the proposed metamaterial resonator is presented with a vector field diagram for different resonant frequencies within the operating frequency region. The induced current for the applied transverse electromagnetic field is distributed on the conducting material due to the interaction of the electric field and magnetic field with the metal-dielectric based subwavelength inclusion. The charges inside the conducting material are canceled with others where the opposite charges produce a net electric field opposite from the electric dipole moment. At 1.9 GHz, the surface current in Fig. 9 follows a clockwise direction over the outer metallic split ring, ranges from 400 to 450 A/m, and is strongly induced on the inner spiral conducting layer, ranging from 450 to 950 A/m. At the second resonant frequency, the current on the conducting surface is densely distributed, ranges from 300 to 400 A/m and follows the directional polarisation in the right side through the outer metallic strip and opposite direction through the inner metallic ring. A circulating electric current is induced on the metallic layer due to the strong magnetic coupling of the time varying magnetic field to enhance the magnetic susceptibility. The magnitude of the magnetic dipole moment depends on the surface current density. For the third resonant frequency, the intensity of the surface current is increased near the inner metallic split ring and spiral metallic arm. At fourth resonant frequency is distributed through the inner spiral type metallic arm.

## Experimental results

**Figure 10:**
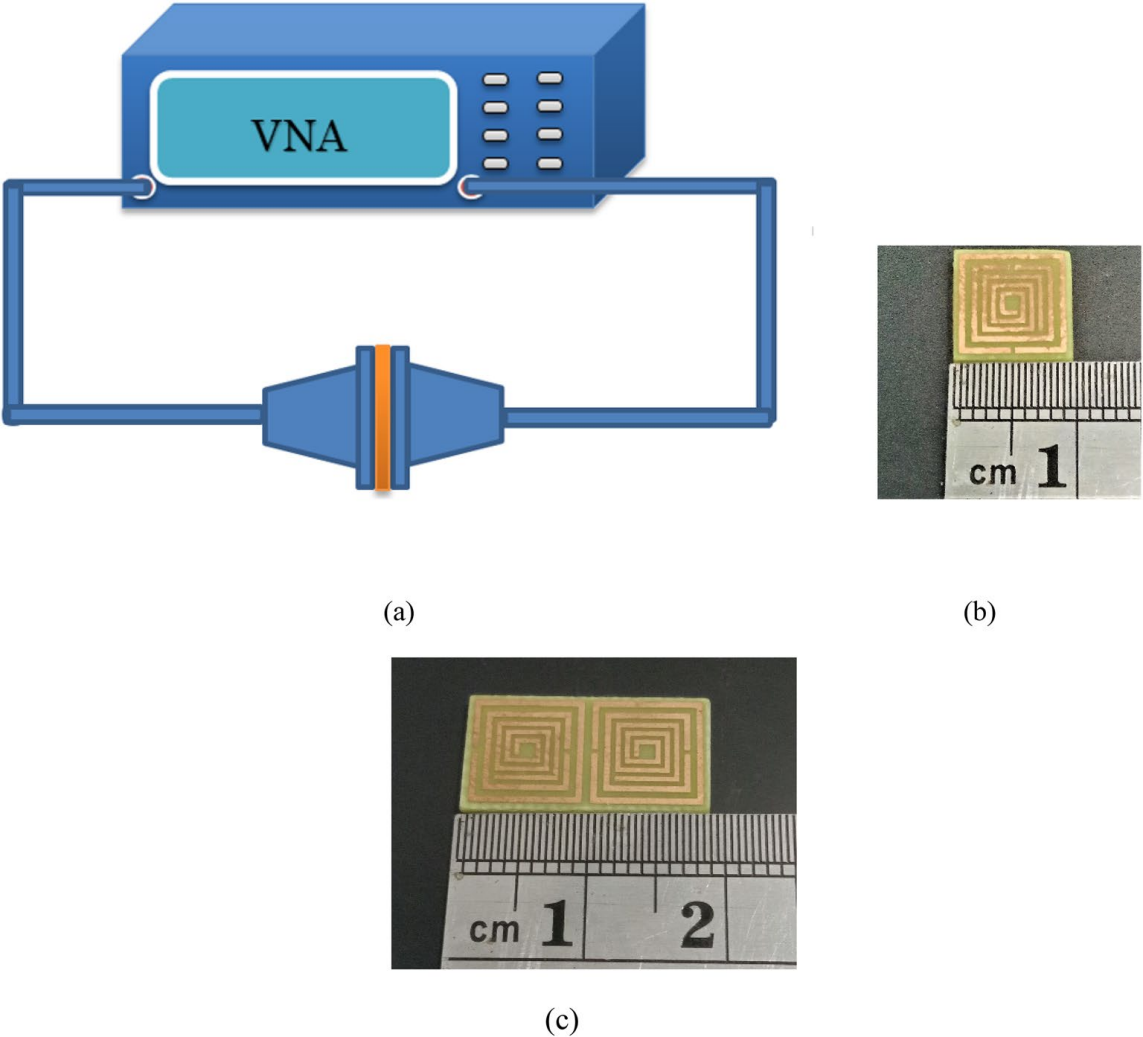
(**a**) Experimental setup and fabricated prototype (**b**) unit cell and (**c**) 1 × 2 array cell.

**Figure 11:**
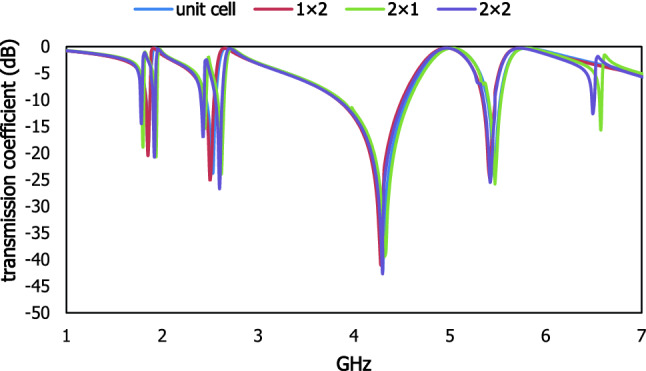
Scattering properties of different array cells.

**Figure 12:**
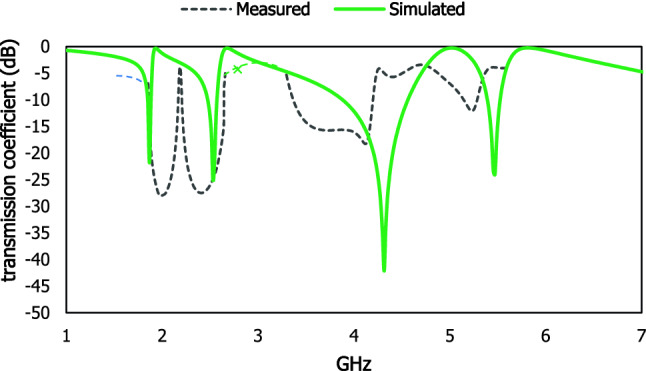
Simulated and measured result of the proposed design.

The interaction between the applied electric field and magnetic field attributes unique properties for the characterization of the materials scattering properties in the microwave frequency region. For the experimental measurement in Fig. 10, a vector network analyser with the peripheral devices such as waveguide port, connector, the cable is used to extract scattering properties of the proposed metamaterial resonator for different frequency ranges. The metallic layer is mechanically etched on the dielectric material FR-4. The simulated result for different array cells is presented in Fig. 11. The unit cell and 1 × 2 array cell are shown similar scattering properties, where the 2 × 1 and 2 × 2 array cell exhibit discrepancies with the results of transmission coefficient due to the mutual coupling between the cut wire portion of the outer split ring resonator and the continuous metallic arm of the two column-wise unit cells. The simulated and experimental results in Fig. 12 are shown dissimilarities due to some reasons such as the defect in the dielectric lattice structure, changing dielectric constant due to intra-space between the atoms, leakage of EM signal, fabrication process, etc. Moreover, two resonant frequencies in the S-band exhibit wider bandwidth due to the number of sampling in the vector network analyzer and the amplitude of dB of two resonant frequencies in the C band decreases for the effect of skin depth in the fabricated prototype within high-frequency region. The mutual resonance effect at transmitting and receiving end, fabrication error, crystal defect which can change the permittivity of the substrate material and equivalent capacitance value of the system.

## Parametric analysis

**Figure 13:**
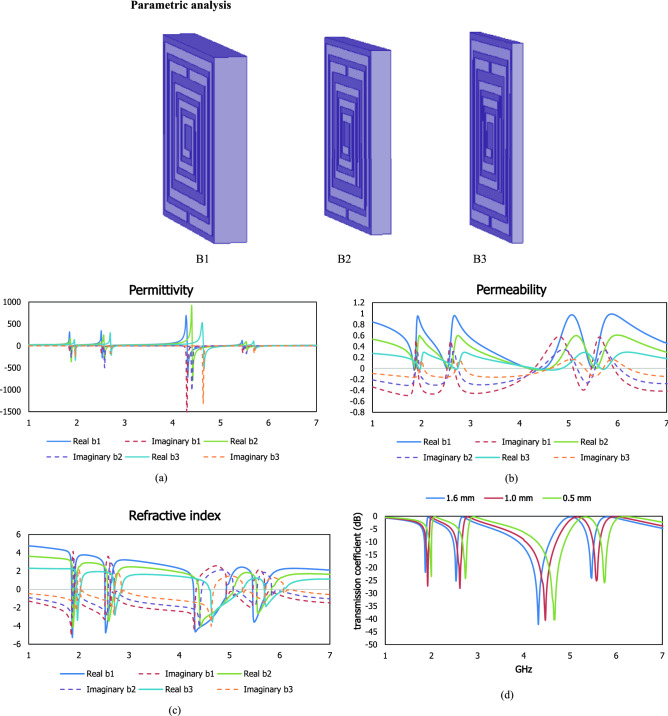
Relative permittivity (**a**), relative permeability (**b**) and refractive index (**c**) and transmission coefficient (**d**) for different thicknesses.

The skin depth of the dielectric material for the interaction of the transverse electromagnetic wave in a higher frequency region is related to the scattering properties of the subwavelength based resonator with the layered conducting surface. The response of effective parameters in Fig. 13 is presented for different thicknesses such as 1.6 mm for design B1, 1.0 mm for design B2 and 0.5 mm for design B3. For the first resonant frequency, the metamaterial unit cell exhibits a small deviation within the lower wavelength region for different thicknesses. The intensity of the real and imaginary values of the relative permittivity over free space is varied with the reduction of the thickness of the dielectric substrate. For 1.6 and 0.5 mm thickness, the imaginary values exhibit higher intensity than the 1.0 mm slab, where design B2 shows a higher amplitude of the real permittivity value. The high intensity in real negative permittivity has increased the transparency through the metamaterial based resonator and exhibits the opposite sign of the excitation field in the material with the applied field. The deviation between the obtained four resonant frequencies within 1–7 GHz is increased with the reduction of the wavelength of the applied source and the deduction in the imaginary amplitude of permittivity is addressed with the reduction of the dielectric losses.

Time varying magnetic field of the imposed oscillating electromagnetic field produces negative effective permeability for the periodic arrangement of the split ring resonator. The permeability can be changed with the variation in thickness, geometrical orientation and frequency of the applied magnetic field. Real negative permeability is observed in response to the first resonant frequency with different thicknesses, where the negative magnitude of the permeability is increased with the reduction of the homogenous dielectric slab and the positive magnitude of permeability is decreased. To the higher frequency region, the negative permeability region at different resonant frequencies within 1–7 GHz is shifted due to the reduction of the magnetic dipole moment density. A negative refractive index can be obtained due to the subwavelength dimension with tailoring periodical geometric metallic layer and the cut wire system. For the proposed metamaterial resonator, the negative refractive index in Fig. 13 is exhibited to the lower frequency region for the combination of the split ring and spiral shaped conducting layer. The negative refractive index region is a shift to the higher frequency region for the reduction in thickness.

**Figure 14:**
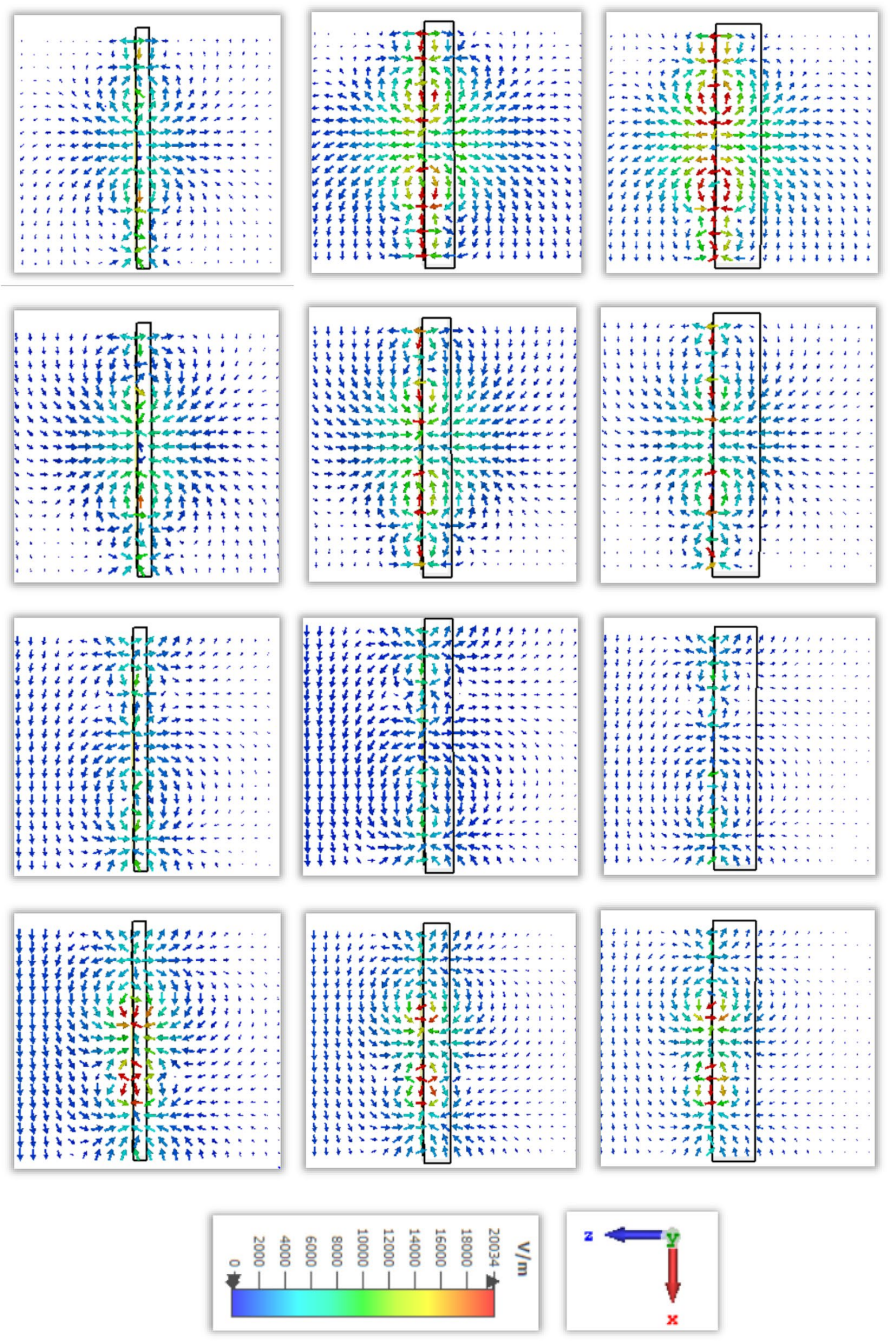
Electric field distribution for different thickness in row-wise ascending from the top order for 1.87, 2.55, 4.32, 5.46 GHz successively.

The distribution of the electric charges migration and dipole orientation in a homogeneous dielectric medium is related to the presence of the applied electric field. The electric field distribution for four resonant frequencies with different thicknesses is shown in Fig. 14. For the first resonant frequency, the interaction of the external electric field through the dielectric substrate gets polarised along the horizontal plane. The adjacent area of this metamaterial resonator exhibits the strength of the electric field polarisation over free space, where the higher volumetric dielectric material exhibit more intense than the lower thicknesses of dielectric material. For the thickness of 1 mm, the polarisation over free space covers more area. For the second resonant frequency, the polarisation strength is exhibited adjoining part of the conducting layer than the dielectric material. For the third resonant frequency, the electric field polarisation around the inner split ring resonator and the direction to the outward from the middle part of the metamaterial resonator. For the fourth resonant frequency, the electric field polarisation is occurred around the inner spiral shape conducting layer and exhibit the similar strength for different thickness of the dielectric material.

**Figure 15:**
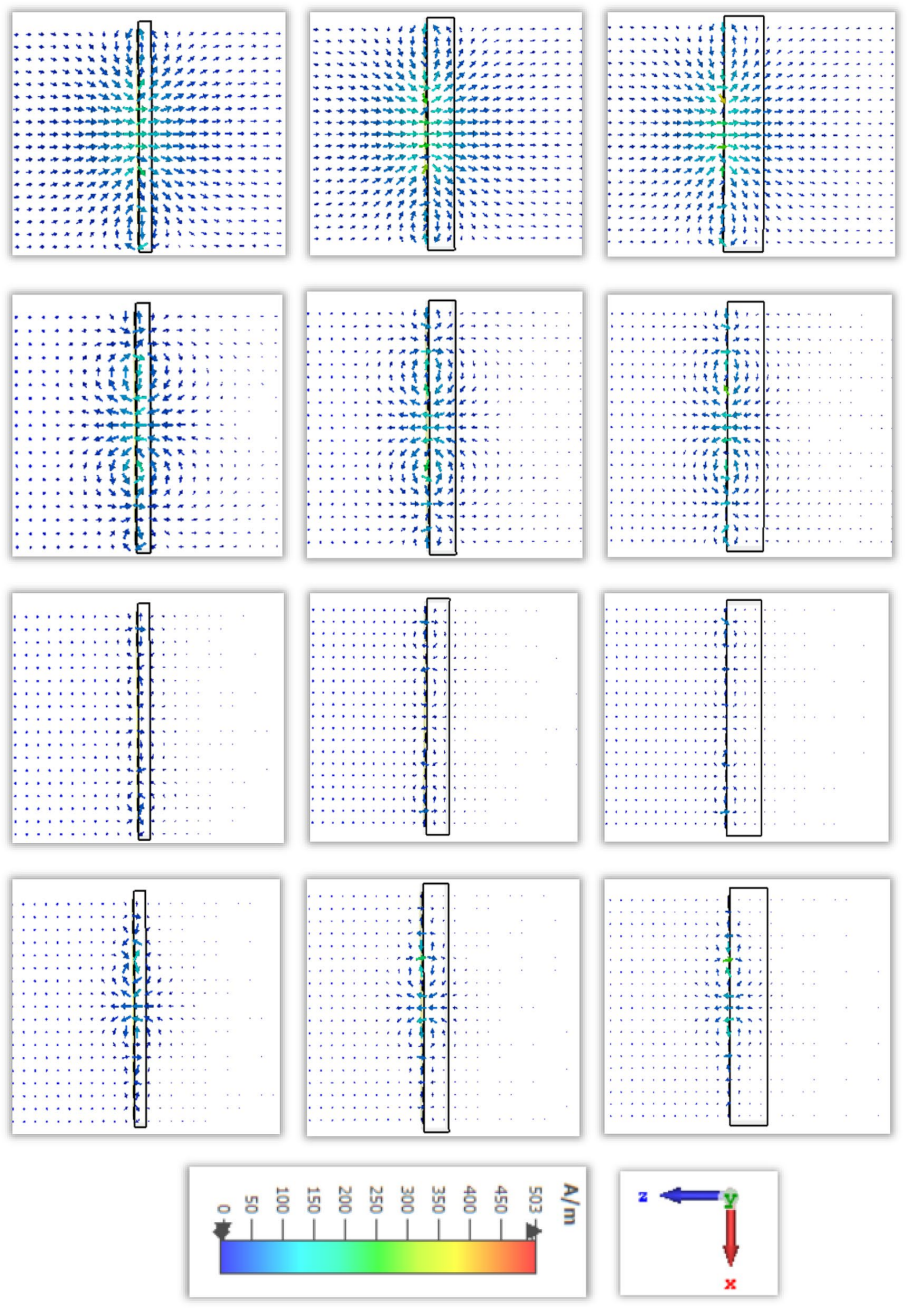
Magnetic field distribution for different thickness in row-wise ascending from the top order for 1.87, 2.55, 4.32, 5.46 GHz successively.

The degree of magnetization of a material is related to the magnetic susceptibility for the imposed magnetic field, where the magnetic dipole moment is produced for the uneven distribution of the electrons and irregular rotation of molecules. The vector field presentation in Fig. 15 for the applied time varying field is exhibited for first resonant frequencies more intense of polarisation over free space near the conducting material. For the second resonant frequency, the magnetic field vector follows the clockwise direction in the upper portion and anticlockwise direction in the lower portion of the resonator. Reducing the dielectric substrate, the intensity for the magnetic polarisation is increased over the free space for the third and fourth resonant frequency. Due to the subtraction of the volumetric dimension which reduces the charge volume density, the magnetic field interaction with the conducting material becomes dominant over the dielectric material.

**Figure 16:**
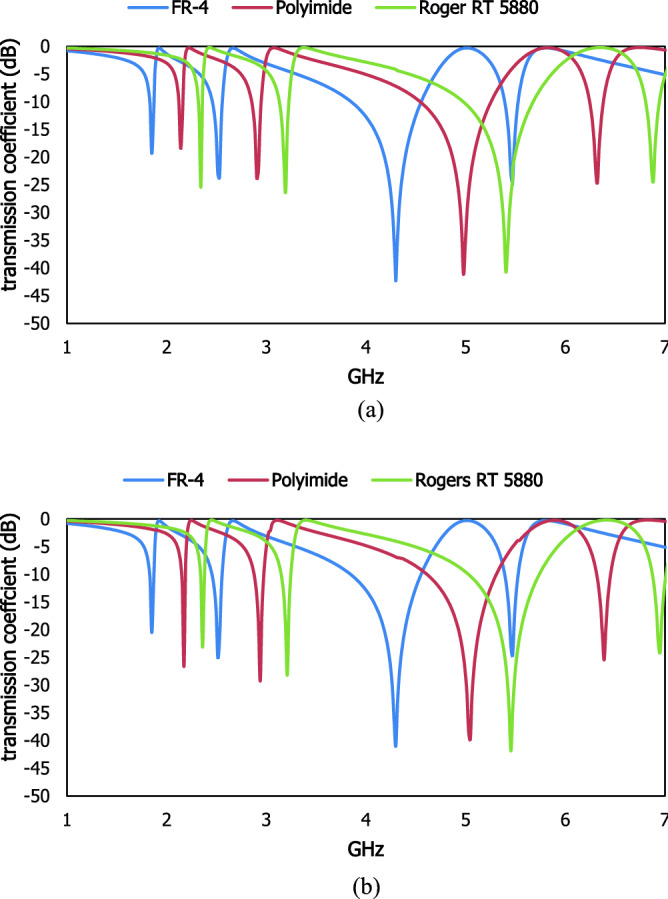
Transmission coefficient results of the different dielectric substrates for the unit cell (**a**) and 1 × 2 array cells (**b**).

With the variation in the value of the dielectric constant, the propagation of an electromagnetic wave through the material will change. The charge centers of the dielectric material are moved from the lattice structure for the external electric field excitation which increases the moment of inertia to produce polarisation. The transmission coefficients are distributed within the operating frequency range exhibit the scattering response for unit cells and 1 × 2 array cells in Fig. 16 with different dielectric constants such as 4.3 for FR-4, 2.9 for polyimide and 2.2 for Rogers RT 5880. The resonant frequencies (1.85, 2.54, 4.3, 5.45) GHz for FR-4, (2.18, 2.87, 5.0, 6.35) GHz for polyimide and (2.35, 3.2, 5.47, 6.96) GHz for Roger RT 5880. For the 1 × 2 array cells resonant frequencies are produced at (1.85, 2.53, 4.3, 5.47) GHz for FR-4, (2.15, 2.91, 4.96, 6.3) GHz for polyimide, (2.35, 3.2, 5.47, 6.94) GHz for Rogers RT 5880. The bandwidths of these resonant frequencies are shown in Table 3. The scattering properties at the transmission end exhibit a similar result between the unit cell and 1 × 2 the array cell which is addressed the low mutual coupling effect with the adjacent metamaterial unit cell and fulfills the periodic boundary condition.

**Table 3:** Bandwidth specification for unit cell and 1 × 2 array cell.

Substrate	Dielectric constant	Bandwidth unit cell (GHz)	Bandwidth 1 × 2 array cell (GHz)
FR-4	4.3	1.83–1.9, 2.48–2.58, 3.84–4.6, 5.4–5.5	1.8–1.89, 2.45–2.57, 3.84–4.57, 5.37–5.55
Polyimide	2.9	2.1–2.22, 2.84–2.3, 4.55–5.26, 6.2–6.4	2.11–2.18, 2.87–2.98, 4.57–5.28, 6.24–6.4
Rogers RT5880	2.2	2.33–2.4, 3.12–3.28, 5.05–5.75, 6.87–7.01	2.31–2.4, 3.11–3.27, 5.03–5.75, 6.85–7.0

**Figure 17:**
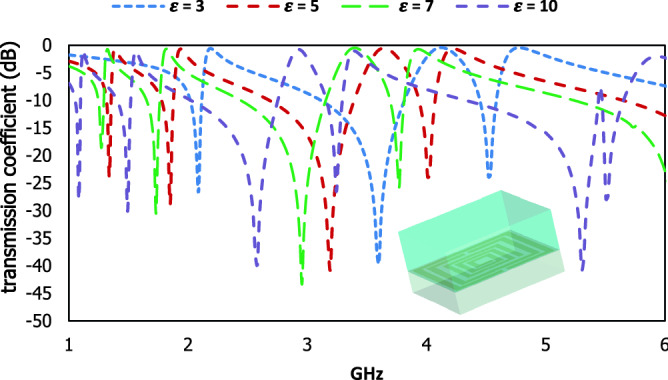
Results of the different dielectric materials as a sample on the proposed resonator.

Different dielectric materials are used on the proposed metamaterial unit cell to characterize the material properties within the functional frequency range. The transmission coefficient for different dielectric constants is presented in Fig. 17. The resonant frequencies are located at 2.1, 3.6, 4.5 GHz for ε = 3, 1.3, 1.83, 3.2, 4 GHz for ε = 5, 1.27, 1.74, 3, 3.8 GHz for ε = 7 and 1.1, 1.5, 2.57, 3.3, 5.34, 5.5 GHz for ε = 10. The polarizability of the material is changed due to the number of bound charges in the dielectric material where the polarisation for the transverse electric field changes the response of the electric dipole moment. When the difference between the dielectric constant of the adjacent sample material with the proposed design is increased, the deviation of the resonant frequencies also increased.

**Figure 18:**
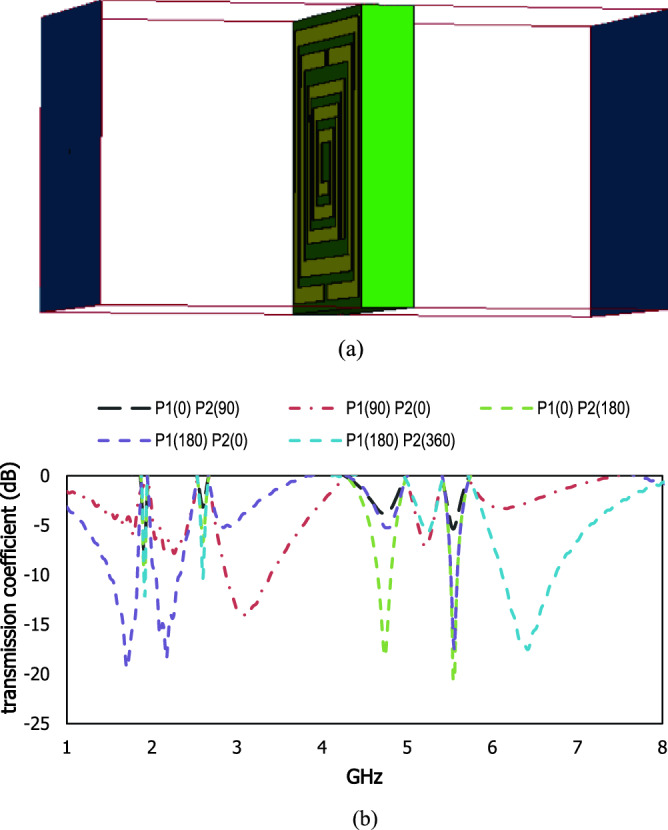
(**a**) Two waveguide ports and (**b**) scattering results for different phase responses in two ports.

This proposed square shaped metamaterial unit cell exhibit reconfigurability with the phase shifting of the applied electromagnetic wave. The scattering properties are changed with variation of phase difference in the two ports and the resonant frequencies are shifted to the different frequency region which is shown in Fig. 18. The phase difference in port 2 with 90 degrees shows the irregular responses, whereas the same phase difference in port 1 exhibits the resonant frequency with wider bandwidth at 3.2 GHz. Two resonant frequencies of transmission coefficient are shown in S-band and other two resonant frequencies in C band for 180 degrees phase difference in port 2, where the resonant frequencies with wider bandwidth are produced in S-band for 180 degrees phase difference in port 1. To increase the phase difference in port 2 by 180 degrees, the resonant frequency in the C band is produced with a wider bandwidth. The variation in the phase response of the applied energy source shows the reconfigurability of the proposed design.

In this context, metamaterial inspired subwavelength based designs are continued their progress in modern wireless communication technologies and the contribution of some researchers in different fields is presented in Table 4. Complementary triangular shaped metallic layer on the FR-4 substrate^31^ with the dimension 8 × 7 mm^2^ was designed for the single negative characteristics to enhance the antenna performance and the effective medium ratio of 6.46. Double H shape^32^ complementary resonator was designed for S-, X-, Ku band with the effective medium ratio of 10.8. A circular split ring resonator^33^ with the dimension 8 × 8 was presented for the 15.4 GHz improving transmission spectrum with the modification of fractal order. Large dimension utilized for the lower microwave frequency region from 600 to 800 MHz was fabricated using rectangular shaped metallic layer and 2 × 2 array cell was printed to provide engineered magnetic material for the gain improvement with the effective medium ratio 8.84^34^. Parallel LC shaped resonator^35^ was introduced wider bandwidth at 5.1 and 10 GHz and Aztec shape metamaterial^36^ unit cell with lower effective medium ratio was established for C, X band applications. A decagon shaped split ring based metamaterial^37^ resonator was shown narrow resonance with negative permeability at 5.2 GHz and the dimension was 15 × 15 mm^2^ with the low effective medium ratio of 4. An omega shaped metamaterial^38^ resonator is designed to achieve magnetic resonance for a multiband bowtie based antenna with the dimension 15 × 15 mm^2^. Rogers RT 5880 substrate^38^ with the dimension 9 × 9 mm^2^ was used for the square split ring resonator which covers the C, X band with the effective medium ratio of 13.3. An open loop resonator using 3 × 2 array cells for M|IMO based antenna^40^ was introduced for the C band applications with the effective medium ratio of 10. Using Rogers RT5880 substrate^41^ with the effective medium ratio of 5.8 was designed for WiFI/WiMax and ISM band application.

**Table 4:** Comparison specification with different established metamaterial unit cells.

	Dimension (mm^2^)	Shape	Substrate	Resonant frequency	Effective medium ratio (EMR)
^31^	8 × 7	CTR	FR-4	5.8, 8	6.46
^32^	9 × 9	Double H shape	FR-4	3.1, 10.1, 11.9, 12.4	10.8
^33^	8 × 8	Circular	FR-4	15.4	2.43
^34^	53.2 × 47.5	Rectangular	FR-4	.64	8.84
^35^	10 × 13	Parallel LC	FR-4	5.1, 10	4.52
^36^	11 × 12	Aztec	FR-4	7.1, 11.2	4.1
^37^	15 × 15	DSRR	FR-4	5.2	4
^38^	15 × 15	Omega	FR-4	6.6	3.03
^39^	9 × 9	SSRR	Rogers RT 5880	2.5, 4.7	13.3
^40^	4.8 × 3	Open loop	FR-4	6	10
^41^	9 × 9	Parabolic	Rogers RT 5880	5.8	5.74
Proposed	9 × 9	Hybrid	FR-4	1.87, 2.55, 4.32, 5.46	17

In this research article, we presented a metamaterial resonator with the hybrid configuration of the dual split ring resonator and spiral shape open loop resonator. This geometric configuration of the conducting layer on the dielectric substrate enhances the magnetic flux which induces higher surface current, ranges from 300 to 500 A/m. The engineered metallic layer is exhibited negative effective parameters along with both plane polarisation for the interaction with the time varying electric and magnetic field. The hybrid combination increases the compactness with the subwavelength dimension which makes this metamaterial preferable to deploy in microwave based devices with improved scattering characteristics over free space.

## Conclusion

Two square split rings with spiral shape metallic arms is created a hybrid configuration of geometric layer on the dielectric substrate. This configuration of this proposed resonator exhibits negative permittivity, negative permeability, negative refractive index and enhances the surface current for the applied transverse electromagnetic wave. The reduction in the thicknesses of dielectric material exhibits the intense polarisation over the free space for different resonant frequencies. The electric and magnetic field polarisation occurs in both planes for the different resonant frequencies. The vector presentation of electric field and magnetic field in the Y-plane are shown the strong response of polarisation which enhances the left handed characteristics. The amplitude and the region of negative effective parameters are changed with the variation in dielectric substrate thickness. A high effective medium ratio increases the compactness of the proposed metamaterial unit cell and makes it preferable to integrate with a different type of microstrip-based antenna for different satellite band and composite material sensing applications.
